# Newly Isolated Animal Pathogen *Corynebacterium silvaticum* Is Cytotoxic to Human Epithelial Cells

**DOI:** 10.3390/ijms22073549

**Published:** 2021-03-29

**Authors:** Jens Möller, Anne Busch, Christian Berens, Helmut Hotzel, Andreas Burkovski

**Affiliations:** 1 Microbiology Division, Department of Biology, Friedrich-Alexander-Universität Erlangen-Nürnberg, 91054 Erlangen, Germany; jens.moeller@fau.de; 2 Institute of Bacterial Infections and Zoonoses, Friedrich-Loeffler-Institute, 07743 Jena, Germany; anne.busch@med.uni-jena.de (A.B.); Helmut.Hotzel@fli.de (H.H.); 3 Department of Anaesthesiology and Intensive Care Medicine, Jena University Hospital, 07747 Jena, Germany; 4 Institute of Molecular Pathogenesis, Friedrich-Loeffler-Institute, 007743 Jena, Germany; Christian.Berens@fli.de

**Keywords:** caseous lymphadenitis, diphtheria, diphtheria toxin, host-pathogen interaction, macrophage, Vero cells, zoonosis

## Abstract

*Corynebacterium silvaticum* is a newly identified animal pathogen of forest animals such as roe deer and wild boars. The species is closely related to the emerging human pathogen *Corynebacterium ulcerans* and the widely distributed animal pathogen *Corynebacterium pseudotuberculosis*. In this study, *Corynebacterium silvaticum* strain W25 was characterized with respect to its interaction with human cell lines. Microscopy, measurement of transepithelial electric resistance and cytotoxicity assays revealed detrimental effects of *C. silvaticum* to different human epithelial cell lines and to an invertebrate animal model, *Galleria mellonella* larvae, comparable to diphtheria toxin-secreting *C. ulcerans.* Furthermore, the results obtained may indicate a considerable zoonotic potential of this newly identified species.

## 1. Introduction

Of the about 130 species [1] comprising the genus *Corynebacterium (C.)*, more than half were isolated from animal and human sources or clinical material indicating a considerable pathogenicity potential of the genus [2]. Moreover, different corynebacteria can be lysogenized by *tox* gene-carrying corynephages [3] and as a result may produce the extremely potent diphtheria toxin (DT). Based on this characteristic, the species *Corynebacterium diphtheriae*, *Corynebacterium ulcerans* and *Corynebacterium pseudotuberculosis* were clustered together in the group of toxigenic corynebacteria [4]. The most prominent member of these putatively toxin-secreting bacteria is *C. diphtheriae*, the type strain of the genus and etiological agent of diphtheria. Interestingly, today diphtheria cases in Western Europe are more frequently connected to infections by *C. ulcerans*, a closely related zoonotic pathogen, which was isolated from a case of diphtheria-like illness already in 1927 [5] and is considered an emerging pathogen today [6,7].

*C. ulcerans* is often found as a commensal of mammals, which may serve as reservoir for zoonotic transmission [8,9,10,11]. With isolates reported from camels, cats, cattle, dogs, foxes, goats, ground squirrels, hedgehogs, monkeys, orcas, otters, owls, pigs, platypus, roe deer, shrew-moles, water rats and wild boars, the host spectrum of *C. ulcerans* is extremely broad [7,12]. Based on the rising number of human infections, this species is recognized as an emerging pathogen [13]. Even nematodes and wax moth larvae can be colonized successfully and have been used as invertebrate model systems to investigate pathogenicity determinants of this species [14,15,16,17,18].

*C. pseudotuberculosis* is an important animal pathogen, which predominantly infects small ruminants, but has also been observed as a pathogen of other farm animals, e.g., cattle and horses [19]. The bacterium is a significant cause of morbidity in sheep and goats and caseous lymphadenitis in these animals leads to significant economic losses in meat, milk and wool production [20].

In 2009, two putative *C. ulcerans* strains were isolated from wild boars with caseous lymphadenitis in Germany, which could not be unequivocally differentiated from *C. pseudotuberculosis* [21]. The isolates were grouped as porcine cluster [21]. Later, another *C. ulcerans* strain was added to this cluster by Rau and co-worker [22]. The following characterization revealed that the isolates within this cluster are non-toxigenic *tox* gene-bearing (NTTB) bacteria and are positive for the secretion of phospholipase D (PLD) [22]. The cluster was extended with further *C. ulcerans* isolates in a study by Berger and co-workers [12] and entitled as the NTTB wildlife cluster. The first designation of the novel species *C. silvaticum* was introduced by Dangel and collaborators [23]. The species currently comprises 38 strains, including isolates from Germany, Austria and Portugal [23,24,25,26].

Recently, the genome sequence of an atypical *C. ulcerans* strain was published [27]. Strain W25 was isolated from a wild boar shot during a hunt near Ilfeld, Thuringia, Germany. The male piglet had an age of approximately 11 months and a bad nutritional status with a body weight of only 16 kg. Hairless regions at chest and flanks gave rise to the suspicion of mange, which could, however, not be confirmed by detection of mites. Lymph nodes at head, neck and groin (*Lymphonodi parotidei*, *Lymphonodi cervicales*, *Lymphonodi inguinales*) were swollen and purulent and showed necrotic lesions, the typical symptoms of necrotizing lymphadenitis. W25 was isolated from the abscesses, while other pathogens like *Salmonella*, yeasts, dermatophytes, different viruses and *Trichinella* parasites were not detected by standard methods. When genome sequence data of isolate W25 were generated, assembled and analyzed, a close taxonomical relationship of the strain to *C. pseudotuberculosis* was initially observed [27]. Later, more detailed taxonomic analyses revealed that isolate W25 is a member of a newly defined species, *Corynebacterium silvaticum*, which mainly infects forest animals such as roe deer and wild boars [23,25,26].

Since we are interested in the virulence of pathogenic corynebacteria [16,28,29,30,31,32,33,34], we started to characterize the newly identified *C. silvaticum* strain W25 with respect to its zoonotic potential. Recent genome analyses indicated a considerable number of virulence factors [26,35] and in proteome analyses a number of pathogenicity determinants were observed already under laboratory conditions [35]. In the study presented here, interaction of *C. silvaticum* strain W25 with human epithelial cell lines was characterized. The results obtained revealed significant cytotoxicity of W25, which is comparable to diphtheria toxin-secreting *C. ulcerans*, and may indicate a zoonotic potential of the newly isolated pathogen.

## 2. Results

### 2.1. In Vitro Colonization of Epithelial Cells by W25

**Figure 1:**
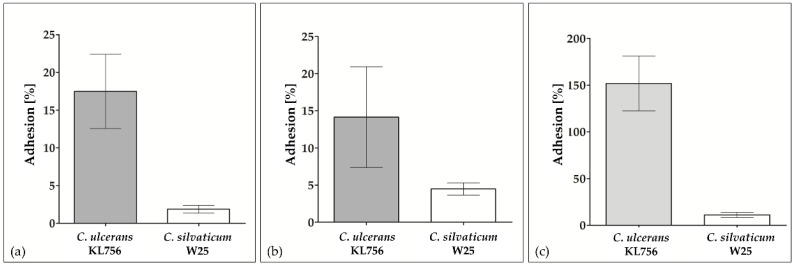
Colonization of epithelial cells. Adhesion of the toxigenic *C. ulcerans* strain KL756 and the non-toxigenic *C. silvaticum* isolate W25 to different human epithelial cell lines: (**a**) Detroit 562 cells, (**b**) HEK-Blue 293 hTLR2 cells, (**c**) HeLa cells. The respective cell line was seeded 24 h prior to infection and infected with bacteria at a multiplicity of infection (MOI) of 50 for 90 min. Columns and error bars represent the results and standard deviations of three independent biological replicates carried out with three technical replicates each (*n* = 9). Adhesion efficiency was calculated based on the ratio of colony-forming units (CFU) prior to infection and CFU on the lysate plates after infection, multiplied by 100.

Despite the fact that W25 is a non-toxigenic *tox*-bearing strain, which does not secrete diphtheria toxin [26], the infected piglet was in rather poor condition. This observation may hint at a high virulence and we suspected a strong pathogenic potential towards human cells as well. Pathogenic corynebacteria typically adhere to epithelia and skin. Therefore, in vitro adhesion assays were carried out. Interestingly, W25 showed inferior adhesion rates to human epithelial cell lines compared to *C. ulcerans*. In case of the pharyngeal cell line Detroit 562 and HEK cells, the adhesion rates of *C. ulcerans* strains exceeded the rate of W25 by a factor of 5. When HeLa cells were tested, *C. ulcerans* strain KL756 reached adhesion rates of 151.90 ± 29.51% after two hours of incubation, i.e., bacteria were not only able to colonize HeLa cells, but were also growing attached to their surface. In contrast, an adhesion rate of only 11.07 ± 2.65% was measured for strain W25 (Figure 1).

For other corynebacteria such as *C. diphtheriae*, it was shown that adhesion and invasion are not strictly coupled processes [36]. Therefore, invasion of different epithelial cell lines was also tested. Again, in comparison to *C. ulcerans* KL756, *C. silvaticum* W25 showed significantly lower invasion rates with less than 2% for all cell lines tested (data not shown).

Taken together, the poor adhesion and invasion rates did not explain or support a high virulence of *C. silvaticum* strain W25. A possible alternative explanation of the poor status of the piglet infected with strain W25 may be a direct detrimental effect on cells.

### 2.2. Microscopic Inspection of Infected Cells

During the assays described above, cell vitality seemed to be impaired upon infection with *C. ulcerans* and *C. silvaticum*. This observation was investigated in more detail by microscopic techniques.

**Figure 2:**
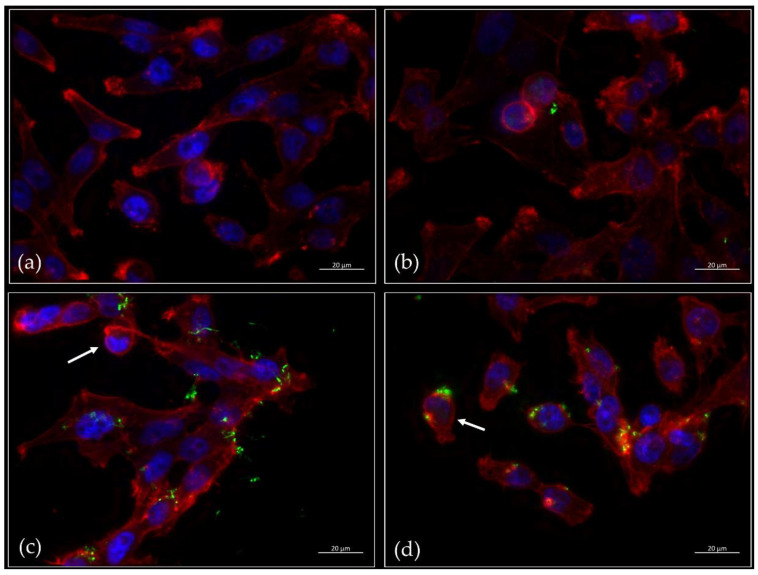
Fluorescence microscopy of epithelial cells infected with different corynebacteria. (**a**) Untreated HeLa cells, cells infected (**b**) with *C. glutamicum* ATCC13032 pERP1p45_*gfp*, (**c**) with *C. ulcerans* KL756 pERP1p45_*gfp* and (**d**) with *C. silvaticum* W25 pERP1p45_*gfp*. Red: Phalloidin-iFluor 647, blue: DAPI, green: GFP. Arrows indicate HeLa cells with morphological changes from spindle to disc-shape forms.

As first approach, HeLa cells were infected with GFP-labelled bacteria and fluorescence microscopy was carried out. In the case of the untreated cells (Figure 2a) and infection with the non-pathogenic *Corynebacterium glutamicum* strain ATCC13032 pERP1p45_*gfp* (Figure 2b), a dense monolayer of spindle-shaped cells attached to the surface was observed. In the case of *C. ulcerans* KL756 pERP1p45_*gfp* (Figure 2c), a partial detachment of cells was seen, which was even pronounced in the case of *C. silvaticum* W25 (Figure 2d). In both cases of pathogenic corynebacteria, morphologic changes from spindle to disc-shaped forms were also observed indicating similar detrimental effects on the HeLa cells.

**Figure 3:**
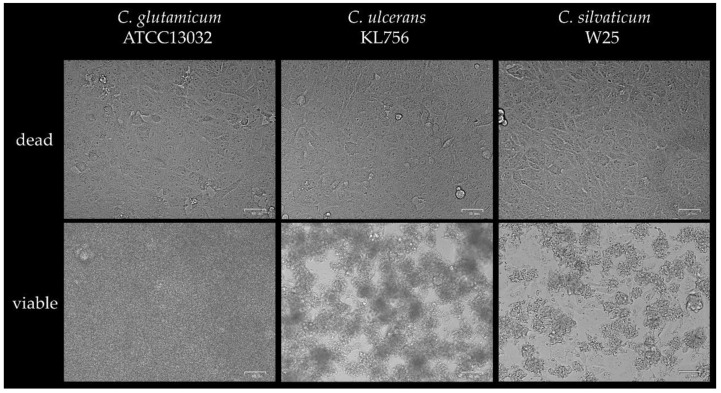
Vero cell assay. Vero cells infected with UV-killed (dead) and viable bacteria at an MOI of 1 after 16 h of incubation under cell culture conditions. Images were done using Bio-Rad Zoe^TM^ Fluorescent Cell Imager.

The gold standard to investigate cytotoxic effects is the Vero cell assay, which was already applied in a modified form to investigate a Shiga-like toxin expressed by *C. ulcerans* and *C. diphtheriae* strains [16]. When Vero cells, a monkey kidney cell line, were infected with UV-killed bacteria at MOI 1 cells showed an intact surface layer of flat spindle-shaped cells. Due to fast and strong bacterial growth, Vero cells were not detectable microscopically after infection with viable *C. glutamicum* ATCC13032. When cells were infected with active *C. ulcerans* KL756 or *C. silvaticum* W25 at an MOI of 1, loss of the even cell monolayer structure and the appearance of rounded cells was observed (Figure 3), further indicating a high cytotoxicity of KL756 and W25. An influence of bacterial growth cannot completely be excluded; however, non-pathogenic *C. glutamicum* showed the highest numbers of bacteria in this assay, followed by *C. ulcerans* KL756. The lowest numbers of bacteria were detected for *C. silvaticum* W25.

### 2.3. Transepithelial Electrical Resistance

**Figure 4:**
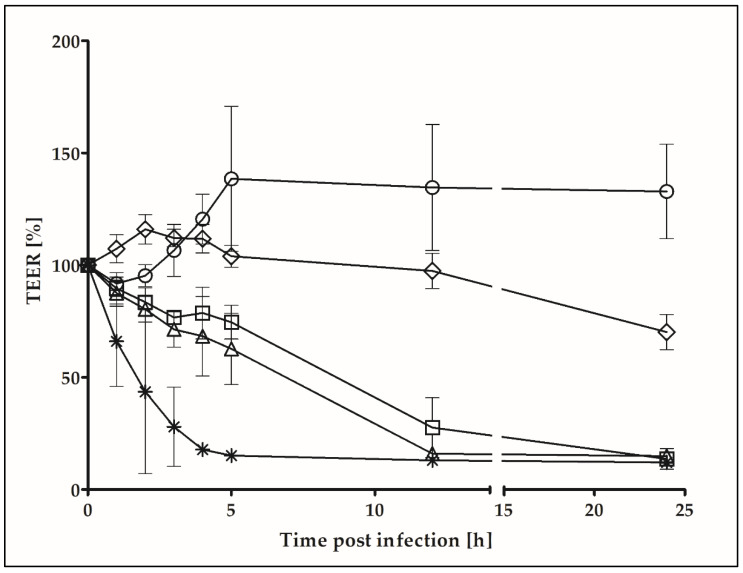
Transepithelial electrical resistance of Detroit 562 cell monolayers. Cells were uninfected (circles) or challenged by *C. glutamicum* ATCC13032 for negative control (rhombi), *Salmonella enterica* serovar Typhimurium NCTC 12023 as positive control (stars), *C. ulcerans* KL756 (triangles) and *C. silvaticum* W25 (squares) with 100 µL bacterial suspension with an OD_600_ of 5. Experiments were carried out as three independent biological replicates, each with three technical replicates and means and standard deviations are shown. Based on the resistance before infection, the relative TEER was calculated for each time point.

Using an alternative and more quantitative approach, cytotoxic effects were determined by measuring the transepithelial electrical resistance (TEER) of polarized cell monolayers. While Detroit 562 cells showed an overall increase in transepithelial resistance during the first 5 h and subsequently a constant resistance for 19 h, DT-secreting *C. ulcerans* KL756 and *C. silvaticum* W25 led to a continuous decrease of transepithelial resistance during the first 12 h of infection, resulting in an almost complete breakdown of TEER (Figure 4). Cells infected with the non-pathogenic *C. glutamicum* strain ATCC13032 showed only a slight decrease in transepithelial resistance until 12 h post infection. After 24 h infection, the wells showed strong bacterial growth, acidified medium and a reduction of the transepithelial electrical resistance to 70.2 ± 7.9%. Deduced from this experiment, *C. silvaticum* W25 is as detrimental to cells as diphtheria toxin-producing *C. ulcerans* and *C. glutamicum* shows, due to growth effects, after 24 h a decrease in TEER.

### 2.4. Recognition of Infection with Corynebacteria by Host Cells

Recently published experiments showed a recognition of infection with *C. diphtheriae* by TLR2 and TLR9 [34]. Therefore, TLR receptor activation was monitored using corresponding HEK reporter cell lines to study the recognition of W25 by human cells.

**Figure 5:**
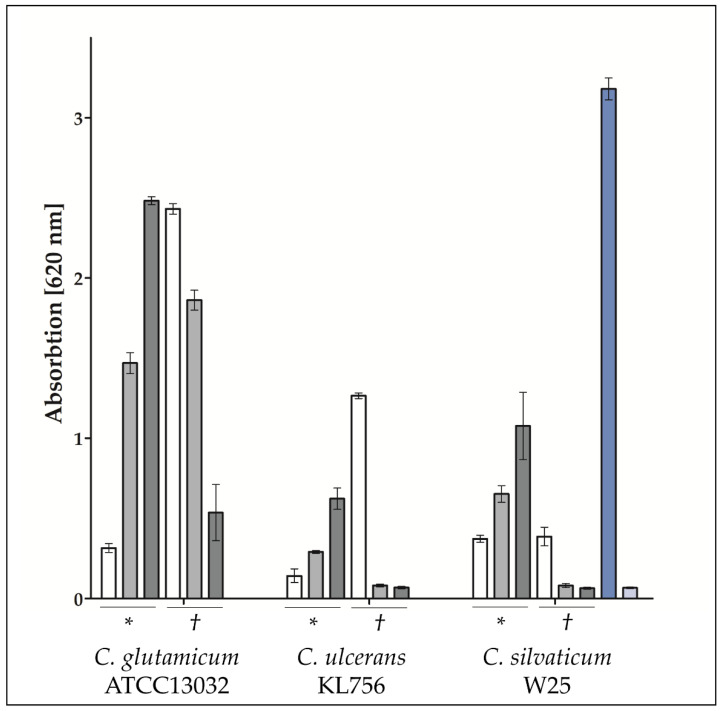
TLR2 receptor activation by *C. glutamicum ATCC13032, C. ulcerans KL756* and *C. silvaticum W25*. HEK-Blue 293 hTLR2 receptor cells were infected at a MOI of 100 (white), 10 (light grey) and 1 (dark grey) with viable (*) and UV-killed (†) bacteria. Heat-killed *Listeria monocytogenes* (HKLM) (blue) were used as positive and phosphate-buffered saline (PBS) (light blue) as negative control. Experiments were performed in three independent biological replicates each in technical triplicates and mean values and standard deviations are shown.

In fact, in case of an infection by *C. glutamicum* ATCC13032, *C. ulcerans* KL756 and *C. silvaticum* W25, TLR2 activation was also observed (Figure 5). Interestingly, when challenged with a high MOI of 100 of viable bacteria, reporter cells showed only a weak TLR2 response, while activation increased from MOI 10 to MOI 1, which indicates that either high numbers of bacteria are detrimental to the reporter cells or TLR activation is actively suppressed by the bacteria. In the case of UV-killed bacteria, TLR2 was activated in a dose-dependent manner with increasing signal strength from MOI 1 to MOI 10 and MOI 100 (Figure 5). No activation was observed for TLR9 (data not shown).

**Figure 6:**
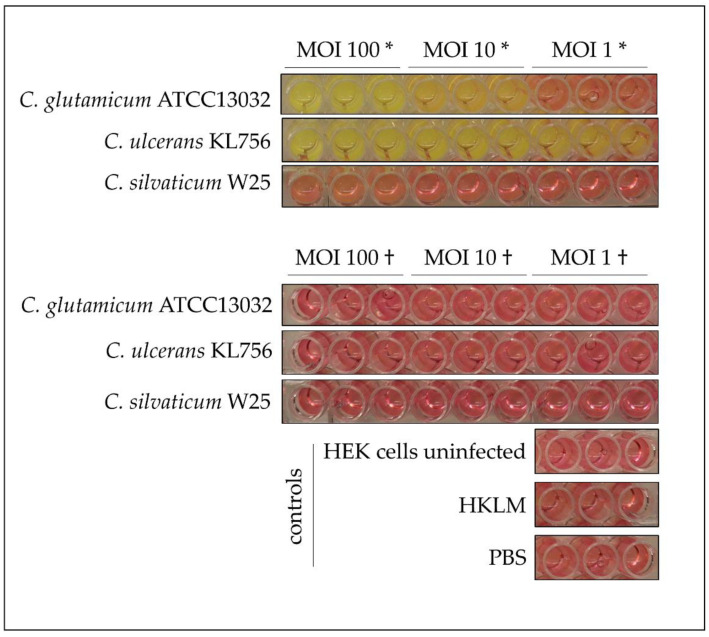
HEK 293 hTLR2 reporter cell assay. Cells were infected with viable (*) and UV-killed (†) bacteria at MOI 100, MOI 10 and MOI 1 for 16 h. Heat-killed *L. monocytogenes* (HKLM) served as positive and PBS as negative control.

To exclude a putative indirect effect of bacterial growth on TLR2 receptor activation, culture medium color was monitored. Phenol red served as an indicator for acidified, spent medium, and changes from red to yellow were correlated with strong bacterial growth, as seen for *C. glutamicum* ATCC13032 at MOI 100 to MOI 10 and *C. ulcerans* KL756 from MOI 100 to MOI 1. No color change was recognized when cells were incubated with UV-killed bacteria, PBS, HKLM and in the case of uninfected cells. In addition, no color change was recognized, when cells were incubated with *C. silvaticum* W25, which may indicate minor or no bacterial metabolism and growth in cell culture medium (Figure 6).

### 2.5. Galleria mellonella Infection Assay

**Figure 7:**
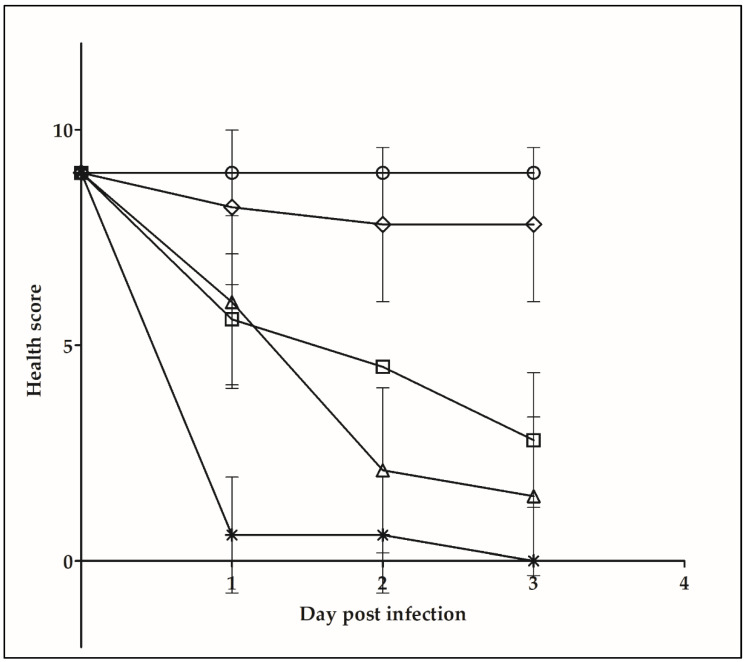
Health score index of *G. mellonella* larvae. Health scores were determined prior and 24, 48 and 72 h after infection. Cycles: MgSO_4_ injection used as a negative control; stars: *Salmonella enterica* serovar Typhimurium NCTC 12023 used as positive control; rhombi: injection of *C. glutamicum* ATCC13032; triangles: injection of *C. ulcerans* KL756; squares: injection of *C. silvaticum* W25.

In addition to the in vitro experiments described above, an invertebrate infection model system was applied, which is suitable for characterization of bacterial pathogens in general [37,38,39] and was already successfully tested for the characterization of pathogenic corynebacteria [15,16]. In accordance with the TEER measurements described above, the NTTB strain W25 showed similar detrimental effects as DT-producing KL756, while *C. glutamicum* had no effect (Figure 7).

## 3. Discussion

In this study, we examined the zoonotic and virulence potential of *C. silvaticum* W25, a recently described species isolated from roe deer and wild boars [23,26,27], which may serve as a reservoir for human infections. As basic pathogenicity parameters, adhesion, invasion and cytotoxicity were characterized.

Adhesion of corynebacteria to host cells is a multifunctional process with pili of *C. diphtheriae* being the best investigated adhesion factors [36]. Pilus expression and adhesion efficiency are correlated in *C. diphtheriae* [36,40] and are important for cell line-dependency of adhesion. For example, *spaA* pili are crucial for adhesion to human pharyngeal carcinoma cells [41]. Pili may also be the reason for the low adhesion rates of *C. silvaticum* W25 to different epithelial cell lines observed in this study. A phylogenetic study by Möller and co-workers [26] revealed a lack of *spaA* and two truncated *spaBC* and *spaDEF* gene clusters, which were suspected to be not functional in this strain. The basal level of adhesion measured may be the result of other factors like NanH, the multifunctional membrane protein DIP0733 (A0A5C5EZI6) or DIP1546 (A0A5F0ACF0), which were shown to be involved in adhesion to host cells in other corynebacteria [42]. Adhesion and invasion are not strictly coupled processes; nevertheless, invasion of epithelial cells by *C. silvaticum* strain W25 is extremely low (supplemental material Figure S1) compared to other corynebacteria [31,42].

The release of lactate dehydrogenase (LDH) due to a loss of membrane integrity served as an indicator of a cytotoxic effect to eukaryotic cells. When cytotoxicity to D562 and Vero cells was examined by LDH assays, a strong cytotoxic effect of *C. silvaticum* W25, but a negative value for *C. ulcerans* KL756, was observed, when cells were infected with viable bacteria at a MOI of 1. No cytotoxicity was detected when cells were infected with UV-killed bacteria at the same MOI or incubated with *C. glutamicum* ATCC13032. An incubation of LDH with bacteria and a following LDH assay revealed that viable *C. silvaticum* W25 and *C. ulcerans* KL756 were able to reduce LDH activity (Cs: 40%; Cu: 95%). This effect was not detected when LDH was incubated with UV-killed bacteria, as well as for *C. glutamicum*, indicating an active and strain-dependent process. It has to be further examined whether an inhibition of LDH activity, an enrichment of pyruvate or a deprotonation of NADH+H^+^ is accountable for this effect (data not shown).

Nevertheless, a strong detrimental effect on epithelial cells was detected. The cytotoxic potential of *C. silvaticum* W25 is demonstrated by a breakdown of the transepithelial resistance of infected polarized cell monolayers and in fact no difference was recognized between the toxigenic *C. ulcerans* strain KL756 and the NTTB *C. silvaticum* strain W25, suggesting that cytotoxicity is not coupled to DT expression. This is supported by Western blot experiments showing that DT expression is not active in cell culture media (supplemental material Figure S2 and Table S1). Besides DT, the main virulence factor of *C. ulcerans* is phospholipase D (PLD) [7]. PLD most likely contributes to spread of the bacterium within the host from primary location of infection to secondary infection sites followed by death of inflammatory cells as shown for *C. pseudotuberculosis* [43,44]. The exotoxin acts as a sphingomyelinase degrading ester bond in sphingomyelin components of mammal cell membranes [45], causing an increased vascular permeability resulting in cell lysis [46], and therefore a breakdown of the transepithelial electrical resistance. PLD exotoxin may also act as a major virulence factor in *C. silvaticum* [26].

Recognition of multiple pathogens via pathogen-associated molecular patterns (PAMPs) occurs by Pattern Recognition Receptors (PRRs) located on the surface of human cells [47]. Toll-like receptors (TLRs) are integral transmembrane receptors, located on the cell surface, and play a crucial role in activating host defense mechanisms to prevent or fight off infections. TRL2 receptors are activated by peptidoglycan, lipomannan, lipoarabinomannan or lipoproteins and initiate MyD88-dependent and -independent signaling pathways [48,49]. In the initial step, TLR2 forms heterodimers with TLR1 or TLR6 and activates an intracellular signaling pathway, which results in NFкB and AP-1 induction. NFкB activates gene transcription and production of inflammatory cytokines; whereas AP-1 influences transcription of inflammatory genes and the stability of their mRNA [48,50]. TLR2 reporter cell lines infected with UV-killed bacteria revealed a MOI-dependent TLR2 receptor activation, with highest SEAP induction at a MOI of 100. Inverse results were obtained after infection with viable bacteria, where cells infected with MOI 1 showed the highest SEAP production and MOI 100 low SEAP production. This indicates a detrimental effect to epithelial cells due to viable bacteria, which was also observed in infections studies with *C. diphtheriae* strains [34]. Non-pathogenic *C. glutamicum* strain ATCC13032 showed also an effect to HEK293 hTLR2 cells. Decreasing SEAP release from MOI 1 to MOI 100 was detected when cells were infected with viable bacteria. When the plates were analyzed 16 h post infection, strong growth of *C. glutamicum* ATCC13032 and *C. ulcerans* KL756 was detected, while wells incubated with *C. silvaticum* showed no turbidity or color change of medium, suggesting reduced bacterial growth. This indicates an influence to SEAP release in case of strong bacterial growth. Nevertheless, the decreasing SEAP release with increasing MOI of viable *C. silvaticum* W25 cannot be explained by this effect, suggesting a direct detrimental effect to the cells.

TLR9 is a receptor located to the endosome, activated by A/D-type CpG oligodeoxynucleotide (CpG-A) and triggers MyD88-dependent host signaling cascades to produce type I interferon [51,52,53,54]. TLR 9 receptors play a crucial role in detecting invading pathogens to activate innate immune response. The results from TLR9 receptor activation assay, where no activation was detected, correlates with the low invasion rates of *C. silvaticum* W25. A study of non-toxigenic *C. diphtheriae* showed strain-dependent TLR9 receptor activation, which correlates with invasion effectivity. It was suggested that bacteria with low TLR9 receptor activation were not internalized by cell [34].

In vivo experiments using *Galleria mellonella* as model system showed no significant differences in melanization, immobility and death of wax moth larvae between *C. silvaticum* W25 and toxigenic *C. ulcerans* KL756. Melanization is a defense mechanism of the innate immune system of *G. mellonella* and serves as an indicator for bacterial virulence as shown for Gram-positive and Gram-negative bacteria [15,16,37,38,55]. Melanin leads to encapsulation, coagulation and opsonization of invading pathogens. This process is analogous to abscess formation in mammalian infections [39,56]. A former study by Weerasekeera and co-workers [16] showed only slight impact of *G. mellonella* infected with *C. glutamicum* ATCC13032. An infection with *C. ulcerans* 809 larvae showed similar results after five days post infection compared to *C. ulcerans* KL756 and *C. silvaticum* W25 after three days post infection. The results support the zoonotic and pathogenic potential of this newly described *Corynebacterium* species.

Taken together, *C. silvaticum* strain W25 showed cell line-dependent, but generally low adhesion and invasion rates. Despite lacking DT expression, a strong cytotoxic effect on epithelial cell lines was observed. Although *C. silvaticum* showed reduced growth and impaired metabolism in cell culture medium, the strain had a strong detrimental effect on epithelial cells. In contrast, despite strong growth in cell culture medium, *C. glutamicum* had no effect at least during the first twelve hours of the experiments. Obviously, not overgrowth of cells but an active process is responsible for the observed cytotoxic effect of *C. silvaticum* on epithelial cells. The use of different model systems—cell culture with human and animal cell lines and the in vivo model organism *G. mellonella*—indicated zoonotic potential with probable transmission to other species and risk of infections in humans.

## 4. Materials and Methods

### 4.1. Bacterial Strains and Culture Conditions

Bacteria used in this study (Table 1) were incubated in brain–heart infusion broth (BHI) at 37 °C. Bacterial strains used in this study are listed in Table 1. For infection assays, the bacterial strains were inoculated from an overnight culture to an OD_600_ of 0.1 and grown to an OD_600_ of 0.4 to 0.6. Bacteria were harvested by centrifugation, washed with PBS and adjusted to an OD_600_ of 1 in PBS.

### 4.2. Cell Cultures

Detroit 562 were cultivated in Dulbecco’s Modified Eagle Medium (DMEM), high glucose with l-glutamine and sodium pyruvate (Gibco, Grand Island, NY, USA), containing 120 µg/mL penicillin, 120 µg/mL streptomycin and 10% heat-inactivated fetal calf serum (FCS). HEK-Blue 293 hTLR2 were incubated in DMEM with L-glutamine and 4.5 g/L glucose (Gibco, Grand Island, NY, USA), supplemented with 10% heat-inactivated FCS, 50 U/mL penicillin, 50 µg/mL streptomycin, 100 µg/mL normocin, 100 µg/mL hygromycin, 100 µg/mL zeocin and 10 µg/mL blasticidin. HeLa cells were cultured in DMEM (4.5 g/L glucose, l-glutamine (Gibco, Grand Island, NY, USA)) supplemented with 100 mg/mL gentamicin, 12 mg/mL ciprofloxacin and 10% heat-inactivated FCS. All cells were cultured in a CO_2_ incubator at 37 °C. Cells were passaged at a ratio of 1:10 twice per week.

### 4.3. Adhesion and Invasion Assays

For adhesion experiments, HeLa cells were infected with bacteria (MOI = 50) for 1.5 h, rinsed three times with PBS and subsequently lysed by addition of sterile water. For invasion experiments, cells were additionally incubated for 2 h with gentamicin (100 µg/mL) before lysis. Serial dilutions of cell lysates were plated on BHI agar plates and relative adhesion and invasion efficiency was calculated based on the ratio of colony-forming units (CFU) prior to infection and CFU on the lysate plates after infection, multiplied by 100.

### 4.4. Transepithelial Electrical Resistance (TEER) Measurements

Detroit 562 cells were seeded in transwells (12 mm, 0.4 µm pore size, polyester membrane, 12-well plate, Corning Incorporated Costar) at a density of 1 × 10^5^ cells per well and incubated under cell culture conditions (37 °C, 5% CO_2_ and 95% humidity) until a polarized cell monolayer with a TEER of at least 800 Ω cm^−2^ was formed. For infection, bacteria were grown in 50 mL BHI medium with 10% FCS and 0.05 M Tween 80 until they reached the exponential phase (OD_600_ of 0.4 to 0.6), harvested by centrifugation at 4000× *g*, washed with PBS and adjusted to an OD_600_ of 5. One hundred µL of this suspension were used for infection. Transepithelial resistance was measured with an epithelial Volt/Ohm Meter (World Precision Instruments, Friedberg, Germany) for every 60 min; after 180 min the supernatant of the infected Detroit 562 cells was removed and the cells incubated overnight in fresh DMEM medium without antibiotics to avoid detrimental effects of bacterial growth.

### 4.5. Reporter Assay for TLR Receptor Activation

TLR2 and TLR9 binding by corynebacteria was analyzed by infection of HEK Blue 293 hTLR2 and hTLR9 cells (InvivoGen, San Diego, CA, USA). HEK Blue 293 cells carry a stably integrated NFкB- and AP1-inducible secreted embryonic alkaline phosphatase (SEAP) reporter construct. The assay was carried out according to a protocol published by Weerasekera and co-workers [34]. Cells were seeded in 96-well plates, infected with viable and UV-killed bacteria at MOI values of 100, 10 and 1 and incubated at 37 °C in a CO_2_ incubator. Heat-killed *L. monocytogenes* (HKLM) were used as positive control and PBS buffer as negative control. After an incubation period of 16 h, the 96-well plates were centrifuged (350× *g*, 5 min) and 20 µL of the cell-free supernatant was mixed with 180 µL pre-warmed SEAP detection reagent QUANTI-Blue (InvivoGen, San Diego, CA, USA) according to the manufacturer’s protocol. After a 2 h incubation under cell culture conditions, the absorbance at 620 nm was measured using a microplate reader (TECAN Infinite M nano plus, Männerdorf, Switzerland). HEK Blue 293 hTLR9 cells were infected with MOI 1 and MOI 50 with viable and UV-killed bacteria in HEK Blue detection medium and incubated for 24 h under cell culture conditions. SEAP activity was measured as mentioned above.

### 4.6. Fluorescence Microscopy

For fluorescence microscopy, 5 × 10^4^ HeLa cells per well were seeded on round coverslips in 24-well plates. After 24 h incubation, the cells were infected at a MOI of 10 with GFP-expressing *Corynebacterium* strains for 90 min. Cells were washed 3 times in PBS and fixed with 4% paraformaldehyde in PBS for 20 min at 37 °C. After an additional washing step in PBS, cells were stored at 4 °C until staining. Subsequently, the fixed cells were stained with 30 µL Phalloidin-iFluor 647 reagent (Abcam, Cambridge, UK) diluted 1:1000 in Image-iT FX signal enhancer (Life Technologies) for 90 min in the dark. Cells were washed 3 times with PBS and mounted on glass slides using ProLong Gold Antifade Mountant with DAPI (Life Technologies, Carlsbad, CA, USA) and stored at 4 °C. Fluorescence microscopy was carried out using a Zeiss invert AX10 microscope (Carl Zeiss AG, Jena, Germany) and analyzed using the ZEN 2 (blue edition) software package.

### 4.7. Cytotoxicity and LDH Activity Assay

The release of lactate dehydrogenase (LDH) from infected cells due to a loss of membrane integrity as well as LDH activity were measured using the Cytotoxicity Detection Kit (Roche, Basel, Switzerland) according to the supplier’s protocol. Additionally, cell morphology was observed and photographed using a Bio-Rad Zoe^TM^ Fluorescent Cell Imager (Bio-Rad Laboratories GmbH, Feldkirchen, Germany) microscope. For LDH activity, 2 U/mL LDH in cell culture medium were incubated with viable and UV-killed bacteria for 24 h and for cytotoxicity testing, epithelial cells were infected for 16 h with viable and UV-killed bacteria. For measurement, 100 µL supernatant of both were mixed with the provided catalyst solution and incubated in the dark for 30 min. Absorbance was measured in a plate reader (TECAN Infinite M nano plus, Männerdorf, Switzerland) at 490 nm and 620 nm for wavelength correction.

### 4.8. Galleria Mellonella Infection

Infection of *G. mellonella* larvae were conducted as described before [15]. Therefore, bacteria were cultured as described above. Bacteria were harvested by centrifugation (4500× *g*, 10 min) at an OD_600_ of 0.4 to 0.6, resuspended in 10 mM MgSO_4_ and adjusted to OD_600_ of 10. A 50 µL Hamilton syringe was used to inject 5 µL of bacteria via the hindmost left proleg. Uninfected larvae and larvae injected with 10 mM MgSO_4_ were used as control. For each bacterial strain, five wax moth larvae were infected and incubated at 22 °C. Each infected individual larva was monitored at the day of infection and daily for three days using the Health Index Scoring System [39]. A score between 9 and 10 suggests healthy, uninfected larvae, whereas a lower score indicates impaired health.

## Figures and Tables

**Table 1:** Bacterial strains, plasmids and cell lines used in this study.

**Strain**	**Description/Source**	**Reference**
*C. glutamicum* ATCC13032	Type strain, non-pathogenic	[57]
*C. ulcerans* KL756	Dog (*tox*^+^)	[58]
*C. silvaticum* W25	Wild boar (*tox^+^*)	[27]
*Salmonella enterica* serovar Typhimurium NCTC 12023	Wild-type (identical to ATCC 14028)	National Collection of TypeCultures (Colindale, UK)
**Plasmids**	**Description**	**Reference**
pERP1p45_*gfp*	*gfp*uv, Km^R^, rep, per, T1, T2	[59]
**Cell lines**	**Description**	**Reference**
HeLa	Human cervical carcinoma cells	[60,61]
HEK-Blue 293 hTLR2	Human TLR2/NFкB/SEAP reporter HEK293 cells	Invivogen
HEK-Blue 293 hTLR9	Human TLR9/NFкB/SEAP reporter HEK293 cells	Invivogen
Vero	African green monkey kidney epithelial cells	[62]
Detroit 562	Human hypopharyngeal carcinoma cells	[63]

## Supplementary Materials

The following are available online at https://www.mdpi.com/article/10.3390/ijms22073549/s1, Figure S1: Quantitative analysis of invasion to epithelial cells. Invasion of the toxigenic *C. ulcerans* strain KL756 and the non-toxigenic *C. silvaticum* isolate W25 to different human epithelial cell lines; Figure S2: Diphtheria toxin (Dt) expression in RPMI 1640 cell culture medium; Table S1: Bacteria used for Western Blot analysis.

## Abbreviations

CFU	Colony-forming units
DT	Diphtheria toxin
HEK	Human embryonal kidney
LD	Linear dichroism
LDH	Lactate dehydrogenase
NTTB	Non-toxigenic toxin-bearing
OD	Optical density
SEAP	Secreted alkaline phosphatase
TEER	Transepithelial electrical resistance
TLA	Three-letter acronym

## References

[1] LPSN.dsmz.de. www.bacterio.net/corynebacterium.html.

[2] Tauch A., Sandbote J., Rosenberg E., DeLong E.F., Lory S., Stackenbrandt E., Thompson F. (2014). The Family *Corynebacteriaceae*. The Prokaryotes.

[3] Sangal V., Hoskisson P.A., Burkovski A. (2014). Corynephages: Infections of the Infectors. *Corynebacterium diphtheriae* and Related Toxigenic Species.

[4] Riegel P., Ruimy R., De Brie D., Prkost G., Jehl F., Christen R. (1995). Taxonomy of *Corynebacterium diphtheriae* and related taxa, with recognition of *Corynebacterium ulcerans* sp. nov. nom. rev. FEMS Microbiol. Lett..

[5] Gilbert R., Stewart F.C. (1927). *Corynebacterium ulcerans*: A pathogenic Microorganism Resembling *Corynebacterium diphtheriae*. J. Lab. Clin. Med..

[6] Burkovski A. (2016). Pathogenesis of Corynebacterium diphtheriae and Corynebacterium ulcerans. Hum. Emerg. Re-Emerg. Infect..

[7] Hacker E., Antunes C.A., Mattos-Guaraldi A.L., Burkovski A., Tauch A. (2016). *Corynebacterium ulcerans*, an emerging human pathogen. Future Microbiol..

[8] (2011). RKI Diphtherie: Erkrankung durch toxigene *Corynebacterium ulcerans* nach Katzenkontakt—Fallbericht. Epidemiol. Bull..

[9] Lartigue M.-F., Monnet X., Le Fleche A., Grimont P.A.D., Benet J.-J., Durrbach A., Fabre M., Nordmann P. (2005). *Corynebacterium ulcerans* in an immunocompromised patient with diphtheria and her dog. J. Clin. Microbiol..

[10] Hogg R.A., Wessels J., Hart J., Efstratiou A., De Zoysa A., Mann G., Allen T., Pritchard G.C. (2009). Possible zoonotic transmission of toxigenic *Corynebacterium ulcerans* from companion animals in a human case of fatal diphtheria. Vet. Rec..

[11] Dias A.A.S.O., Silva F.C., Santos L.S., Ribeiro-Carvalho M.M., Sabbadini P.S., Santos C.S., Filardy A.A., Myioshi A., Azevedo V.A., Hirata R. (2011). Strain-dependent arthritogenic potential of the zoonotic pathogen *Corynebacterium ulcerans*. Vet. Microbiol..

[12] Berger A., Dangel A., Peters M., Mühldorfer K., Braune S., Eisenberg T. (2019). Tox -positive *Corynebacterium ulcerans* in hedgehogs, Germany. Emerg. Microbes Infect..

[13] Berger A., Teusch B., Heinzinger S., Sing A. (2018). *Corynebacterium ulcerans*—Ein Emerging Pathogen? Daten des Konsiliarlabors für Diphtherie 2011–2016. Epidemiol. Bull..

[14] Antunes C.A., Clark L., Wanuske M.T., Hacker E., Ott L., Simpson-Louredo L., de Luna M.D.G., Hirata R., Mattos-Guaraldi A.L., Hodgkin J. (2016). *Caenorhabditis elegans* star formation and negative chemotaxis induced by infection with corynebacteria. Microbiology.

[15] Ott L., Mckenzie A., Baltazar M.T., Britting S., Bischof A., Burkovski A., Hoskisson P.A. (2012). Evaluation of invertebrate infection models for pathogenic corynebacteria. FEMS Immunol. Med. Microbiol..

[16] Weerasekera D., Möller J., Kraner M.E., Antunes C.A., Mattos-Guaraldi A.L., Burkovski A. (2019). Beyond diphtheria toxin: Cytotoxic proteins of *Corynebacterium ulcerans* and *Corynebacterium diphtheriae*. Microbiology.

[17] Peixoto R.S., Hacker E., Antunes C.A., Weerasekera D., Alves A., De Oliveira D.S., Martins C.A., Júnior R.H., Burkovski A., Mattos-Guaraldi A.L. (2016). Pathogenic properties of a *Corynebacterium diphtheriae* strain isolated from a case of osteomyelitis. J. Med. Microbiol..

[18] Weerasekera D., Stengel F., Sticht H., Mattos-Guaraldi A.L., Burkovski A., Antunes C.A. (2018). The C-terminal coiled-coil domain of *Corynebacterium diphtheriae* DIP0733 is crucial for interaction with epithelial cells and pathogenicity in invertebrate animal model systems. BMC Microbiol..

[19] Dorella F.A., Pacheco L.G.C., Oliveira S.C., Miyoshi A., Azevedo V. (2006). *Corynebacterium pseudotuberculosis*: Microbiology, biochemical properties, pathogenesis and molecular studies of virulence. Vet. Res..

[20] Baird G.J., Fontaine M.C. (2007). *Corynebacterium pseudotuberculosis* and its role in ovine caseous lymphadenitis. J. Comp. Pathol..

[21] Contzen M., Sting R., Blazey B., Rau J. (2011). *Corynebacterium ulcerans* from diseased wild boars. Zoonoses Public Health.

[22] Rau J., Blazey B., Contzen M., Sting R. (2012). *Corynebacterium ulcerans*-Infektion bei einem Reh (*Capreolus capreolus*). Berl. Munch. Tierarztl. Wochenschr..

[23] Dangel A., Berger A., Rau J., Eisenberg T., Kämpfer P., Margos G., Contzen M., Busse H.-J., Konrad R., Peters M. (2020). *Corynebacterium silvaticum* sp. nov., a unique group of NTTB corynebacteria in wild boar and roe deer. Int. J. Syst. Evol. Microbiol..

[24] Rau J., Eisenberg T., Peters M., Berger A., Kutzer P., Lassnig H., Hotzel H., Sing A., Sting R., Contzen M. (2019). Reliable differentiation of a non-toxigenic *tox* gene-bearing *Corynebacterium ulcerans* variant frequently isolated from game animals using MALDI-TOF MS. Vet. Microbiol..

[25] Viana M.V.C., Profeta R., da Silva A.L., Hurtado R., Cerqueira J.C., Ribeiro B.F.S., Almeida M.O., Morais-Rodrigues F., de Castro Soares S., Oliveira M. (2021). Taxonomic classification of strain PO100/5 shows a broader geographic distribution and genetic markers of the recently described *Corynebacterium silvaticum*. PLoS ONE.

[26] Möller J., Musella L., Melnikov V., Geißdörfer W., Burkovski A., Sangal V. (2020). Phylogenomic characterisation of a novel corynebacterial species pathogenic to animals. Antonie Leeuwenhoek.

[27] Busch A., Möller J., Burkovski A., Hotzel H. (2019). Genome sequence of a pathogenic *Corynebacterium ulcerans* strain isolated from a wild boar with necrotizing lymphadenitis. BMC Res. Notes.

[28] Tauch A., Burkovski A. (2015). Molecular armory or niche factors: Virulence determinants of *Corynebacterium* species. FEMS Microbiol. Lett..

[29] Trost E., Al-dilaimi A., Papavasiliou P., Schneider J., Viehoever P., Burkovski A., Soares S.C., Almeida S.S., Dorella F.A., Miyoshi A. (2011). Comparative analysis of two complete *Corynebacterium ulcerans* genomes and detection of candidate virulence factors. BMC Genom..

[30] Hacker E., Ott L., Schulze-Luehrmann J., Lürmann A., Wiesmann V., Wittenberg T., Burkovski A. (2016). The killing of macrophages by *Corynebacterium ulcerans*. Virulence.

[31] Hacker E., Ott L., Hasselt K., Mattos-Guaraldi A.L., Tauch A., Burkovski A. (2015). Colonization of human epithelial cell lines by *Corynebacterium ulcerans* from human and animal sources. Microbiology.

[32] Bittel M., Gastiger S., Amin B., Hofmann J., Burkovski A. (2018). Surface and extracellular proteome of the emerging pathogen *Corynebacterium ulcerans*. Proteomes.

[33] Subedi R., Kolodkina V., Sutcliffe I.C., Simpson-Louredo L., Hirata R., Titov L., Mattos-Guaraldi A.L., Burkovski A., Sangal V. (2018). Genomic analyses reveal two distinct lineages of *Corynebacterium ulcerans* strains. New Microbes New Infect..

[34] Weerasekera D., Fastner T., Lang R., Burkovski A., Ott L. (2019). Of mice and men: Interaction of *Corynebacterium diphtheriae* strains with murine and human phagocytes. Virulence.

[35] Möller J., Schorlemmer S., Hofmann J., Burkovski A. (2020). Cellular and extracellular proteome of the animal pathogen *Corynebacterium silvaticum*, a close relative of zoonotic *Corynebacterium ulcerans* and *Corynebacterium pseudotuberculosis*. Proteomes.

[36] Ott L., Höller M., Rheinlaender J., Schäffer T.E., Hensel M., Burkovski A. (2010). Strain-specific differences in pili formation and the interaction of *Corynebacterium diphtheriae* with host cells. BMC Microbiol..

[37] Jander G., Rahme L., Ausubel F.M. (2000). Positive Correlation between virulence of *Pseudomonas aeruginosa* mutants in mice and insects. J. Bacteriol..

[38] Wand M.E., Müller C.M., Titball R.W., Michell S.L. (2011). Macrophage and *Galleria mellonella* infection models reflect the virulence of naturally occurring isolates of *B. pseudomallei*, *B. thailandensis* and *B. oklahomensis*. BMC Microbiol..

[39] Tsai C.J.-Y., Loh J.M.S., Proft T. (2016). *Galleria mellonella* infection models for the study of bacterial diseases and for antimicrobial drug testing. Virulence.

[40] Mandlik A., Swierczynski A., Das A., Ton-that H. (2008). Pili in Gram-positive bacteria: Assembly, involvement in colonization and biofilm development. Trend Microbiol..

[41] Mandlik A., Swierczynski A., Das A., Ton-that H. (2007). *Corynebacterium diphtheriae* employs specific minor pilins to target human pharyngeal epithelial cells. Mol. Microbiol..

[42] Ott L. (2018). Adhesion properties of toxigenic corynebacteria. AIMS Microbiol..

[43] Fontaine M.C., Baird G.J. (2008). Caseous lymphadenitis. Small Ruminand Res..

[44] Windsor P.A. (2011). Contol of caseous lymphadenitis. Vet. Clin. NA Food Anim. Pract..

[45] Jorge K.T.O.S., Santos T.M., Tartaglia N.R., Aguiar E.L., Souza R.F.S., Mariutti R.B., Eberle R.J., Arni R.K., Portela R.W., Meyer R. (2016). Putative virulence factors of *Corynebacterium pseudotuberculosis* FRC41: Vaccine potential and protein expression. Microb. Cell Fact..

[46] Guimarães A.D.S., Borges F., Pauletti R.B., Seyffert N., Ribeiro D., Lage A.P., Heinemann M.B., Miyoshi A., Maria A., Gouveia G. (2011). Caseous lymphadenitis: Epidemiology, diagnosis, and control. IIOAB J..

[47] Akira S., Hemmi H. (2003). Recognition of pathogen-associated molecular patterns by TLR family. Immunol. Lett..

[48] Oliveira-nascimento L., Massari P., Wetzler L.M., Lauvau G.S., Einstein A., Brinkmann M.M. (2012). The role of TLR2 in infection and immunity. Front. Immunol..

[49] Greene C.M., Mcelvaney N.G. (2005). Toll-like receptor expression and function in airway epithelial cells. Arch. Immunolgiae Ther. Exp..

[50] West A.P., Koblansky A.A. (2006). Recognition and Signaling by Toll-Like Receptors. Annu. Rev. Cell Dev. Biol..

[51] Honda K., Ohba Y., Yanai H., Hegishi H., Mizutani T., Takaoka A., Taya C., Taniguchi T. (2005). Spatiotemporal regulation of MyD88-IRF-7 signalling for robust type-I interferon induction. Nature.

[52] Costa-Mattioli M., Sonenberg N. (2008). RAPping production of type I interferon in pDCs through mTOR. Nat. Immunol..

[53] Honda K., Taniguchi T. (2006). IRFs: Master regulators of signalling by Toll-like receptors and cytosolic pattern-recognition receptors. Nat. Rev. Immunol..

[54] Meylan E., Tschopp J., Karin M. (2006). Intracellular pattern recognition receptors in the host response. Nature.

[55] Cutuli M.A., Petronio G.P., Venditti N., Di Marco R., Vergalito F., Magnifico I., Pietrangelo L. (2019). *Galleria mellonella* as a consolidated in vivo model hosts: New developments in antibacterial strategies and novel drug testing. Virulence.

[56] Tang H., Tang H. (2009). Regulation and function of the melanization reaction in *Drosophila*. Fly.

[57] Abe S., Takayama K.-I., Kinoshita S. (1967). Taxonomical studies on glutamic acid-producing bacteria. J. Gen. Appl. Microbiol..

[58] Möller J., Kraner M., Sonnewald U., Sangal V., Tittlbach H., Winkler J., Winkler T.H., Melnikov V., Lang R., Sing A. (2019). Proteomics of diphtheria toxoid vaccines reveals multiple proteins that are immunogenic and may contribute to protection of humans against *Corynebacterium diphtheriae*. Vaccine.

[59] Knoppová M., Phensaijai M., Veselý M., Zemanová M., Nešvera J., Pátek M. (2007). Plasmid vectors for testing in vivo promoter activities in *Corynebacterium glutamicum* and *Rhodococcus erythropolis*. Curr. Microbiol..

[60] Gey G.O., Coffman W.D., Kubicek M.D. (1952). Tissue culture studies of the proliferative capacity of cervical carcinoma and normal epithelium. Cancer Res..

[61] Scherer W.F., Syverton J.T., Gey G. (1953). Studies on the propagation in vitro of poliomyelitis viruses. IV. Viral multiplication in a stable strain of human malignant epithelial cells (strain HeLa) derived from an epidermoid carcinoma of the cervix. J. Exp. Med..

[62] Yasumura Y., Kawakita Y. (1963). Studies on SV40 in tissue culture: Preliminary step for cancer research In Vitro. Nihon Rinsho.

[63] Peterson W.D., Stulberg C.S., Swanborg N.K., Robinson A.R. (1968). Glucose-6-phosphate dehydrogenase isoenzymes in human cell cultutes determined by sucrose-agar gel and cellulose acetate zymograms. Proc. Soc. Exp. Biol. Med..

