# Supplementary material for: Newly Isolated Animal Pathogen Corynebacterium silvaticum Is Cytotoxic to Human Epithelial Cells
Source: Int J Mol Sci. 2021 Mar 29;22(7):3549. doi: 10.3390/ijms22073549 (PMC8037504; doi:10.3390/ijms22073549)
Supplement: Supplementary file 1 [file ijms-22-03549-s001.pdf]

Supplemental Material

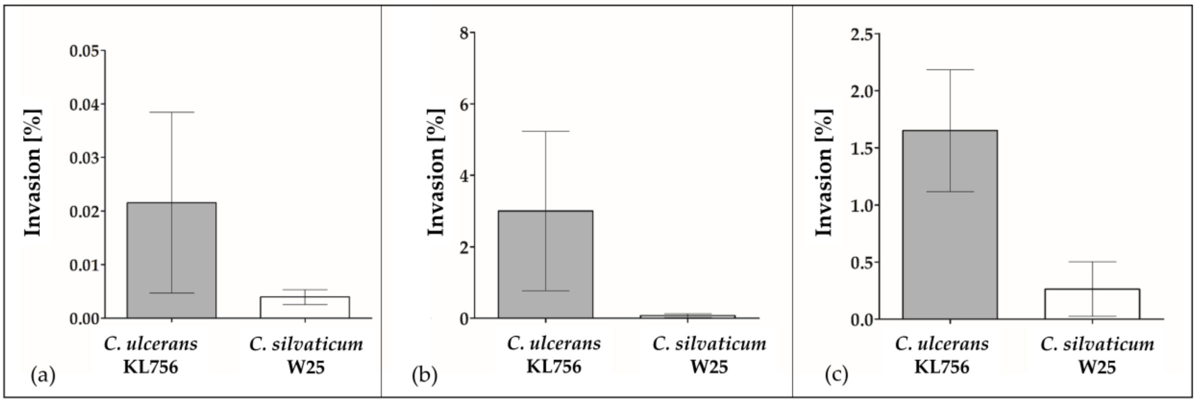

Figure S1. Quantitative analysis of invasion to epithelial cells. Invasion of the toxigenic *C. ulcerans* strain KL756 and the non-toxigenic *C. silvaticum* isolate W25 to different human epithelial cell lines: (**a**) Detroit 562, (**b**) HEK-Blue 293 hTLR2, (**c**) HeLa. The respective cell line was seeded 24 hours prior to infection and infected with bacteria at an MOI of 50. To kill extracellular bacteria, after 90 min post infection the cells were additionally incubated for 2 hours with gentamicin (100 µg/mL) before lysis. Columns and error bars represent the results and standard deviations of three independent biological replicates carried out with three technical replicates each (n = 9). Invasion efficiency were calculated based on the ratio of colony forming units (CFU) prior to infection and CFU on the lysate plates after infection, multiplied by 100.

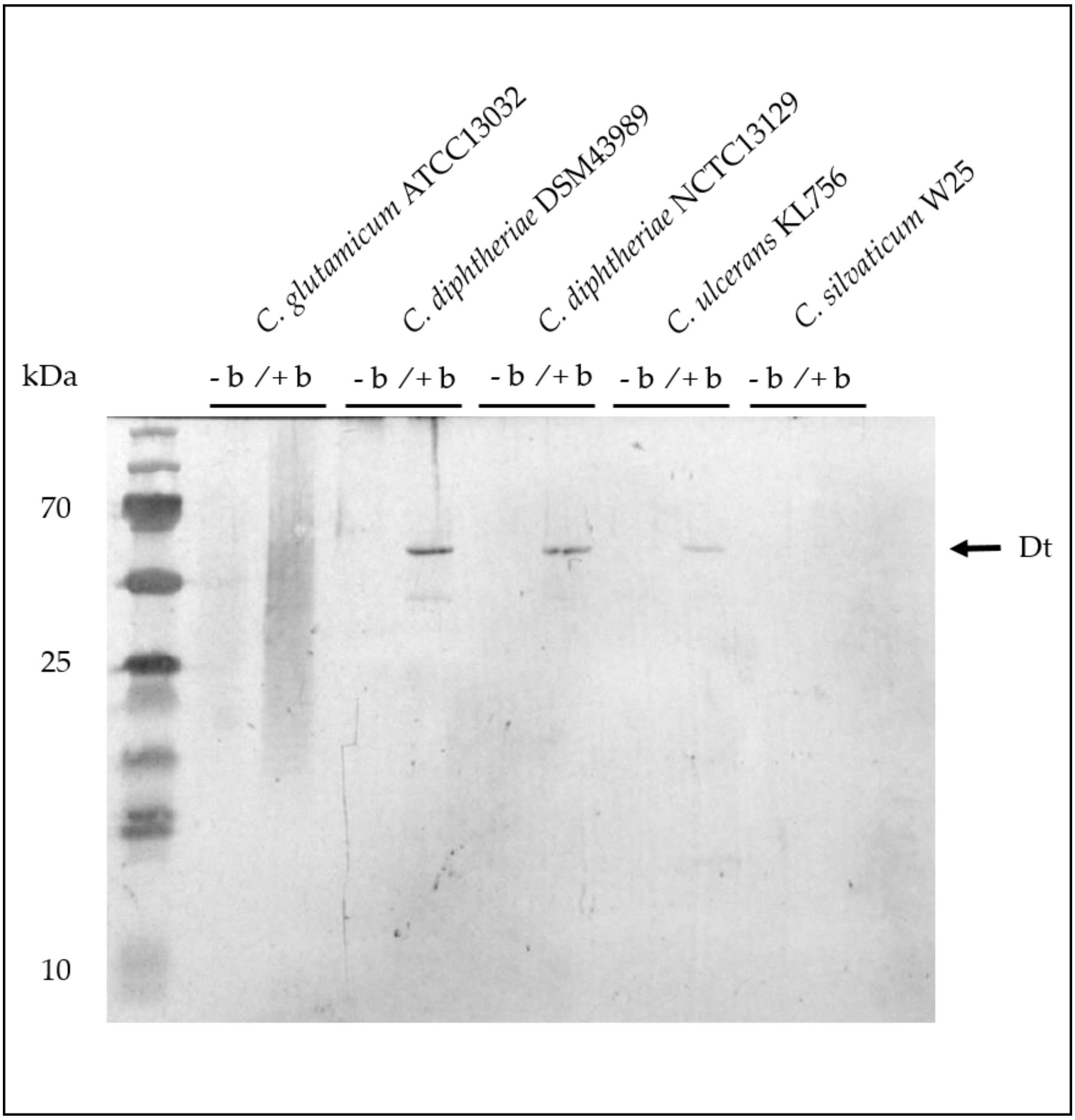

Figure S2. Diphtheria toxin (Dt) expression in RPMI 1640 cell culture medium. The bacteria were incubated without bipyridyl (−b) and Dt expression was induced with 0.05 M bipiridyl (+b). Crude cell extracts were prepared and 40 µg of each sample loaded onto a 1% SDS gel. The separated proteins were transferred to a positively charged PVDF membrane. Detection of the Dt protein was carried out as published previously by Möller and coworkers [1]. Dt has a molecular weight of 62 kDa and is labeled with an arrow.

Table S1. Bacteria used for Western Blot analysis.

Strain	Description/source	Reference
C. ulcerans KL756	Dog (*tox*+)	[1]
C. glutamicum ATCC13032	Type strain, nonpathogenic	[2]
C. diphtheriae DSM43989		[3]
C. diphtheriae NCTC13129	Diphtheria	[4]
C. silvaticum W25	Wild boar (*tox*+)	[5]

References

1. Möller, J.; Kraner, M.; Sonnewald, U.; Sangal, V.; Tittlbach, H.; Winkler, J.; Winkler, T.H.; Melnikov, V.; Lang, R.; Sing, A.; et al. Proteomics of diphtheria toxoid vaccines reveals multiple proteins that are immunogenic and may contribute to protection of humans against *Corynebacterium diphtheriae*. *Vaccine* **2019**, *37*, 3061–3070.
2. Abe, S.; Takayama, K.-I.; Kinoshita, S. Taxonomical studies on glutamic acid-producing bacteria. *J. Gen. Appl. Microbilogy* **1967**, *13*, 279–301.
3. Barksdale, L. *Corynebacterium diphtheriae* and its relatives. *Bacteriol. Rev.* **1970**, *34*, 378–422.

4. Cerdeño-Tárraga, A.M.; Efstratiou, A.; Dover, L.G.; Holden, M.T.G.; Pallen, M.; Bentley, S.D.; Besra, G.S.; Churcher, C.; James, K.D.; De Zoysa, A.; et al. The complete genome sequence and analysis of *Corynebacterium diphtheriae* NCTC13129. *Nucleic Acids Res.* **2003**, *31*, 6516–6523.
5. Busch, A.; Möller, J.; Burkovski, A.; Hotzel, H. Genome sequence of a pathogenic *Corynebacterium ulcerans* strain isolated from a wild boar with necrotizing lymphadenitis. *BMC Res. Notes* **2019**, *12*, 10–12.
